# An EEG Dataset on Aesthetic and Creative Judgments of Brief Structured Poetry

**DOI:** 10.1038/s41597-025-06189-w

**Published:** 2025-12-03

**Authors:** Soma Chaudhuri, Joydeep Bhattacharya

**Affiliations:** 1 Department of Psychology, Goldsmiths University of London, London, UK; 2 Penn Center for Neuroaesthetics, University of Pennsylvania, Pennsylvania, USA; 3 Academy of Music, School of Creative Arts, Hong Kong Baptist University, Hong Kong, China

**Keywords:** Psychology, Cognitive neuroscience

## Abstract

Understanding how the brain engages with poetic language is key to advancing empirical research on aesthetic and creative cognition. We present a 64-channel EEG and behavioural dataset from 51 participants who read and evaluated 210 short English-language texts (70 Haiku, poems focusing on nature; 70 Senryu, poems focusing on emotion; 70 structurally-matched non-poetic control texts). Participants rated each stimulus on five dimensions (aesthetic appeal, vivid imagery, emotional impact, originality and creativity) on a 7-point scale. The dataset includes time-aligned EEG and behavioural responses, self-report trait measures, and rich stimulus metadata. Further, the dataset also includes resting state EEG data before and after the experiment. Exploratory validation analysis revealed condition-specific spectral power differences and trial-level brain-behaviour associations. By combining poetic structure, subjective evaluation, and high-temporal-resolution neural activity, this comprehensive dataset enables detailed investigation into neuroaesthetics, cognitive poetics, and the neuroscience of creativity.

## Background & Summary

Imagine reading Wordsworth’s *The Solitary Reaper*, where the beauty of nature unfolds vividly before your eyes, and the melody of the reaper’s song lingers in your mind. Or turning to Yeats’ *When You Are Old*, where the gentle pull of nostalgia and subtle melancholy fills your heart. These poetic experiences, simultaneously visual, emotional, and rhythmic, are familiar to many readers. Yet, the underlying neurocognitive processes that shape our experience of poetry remain only partially understood^1,2^.

Poetic language is rich in stylistic devices and ‘figures of thought’, such as metaphor, conceit or extended metaphor, polysemy, irony, metonymy, oxymoron and so on, which engage both affective and cognitive processes^3^. Such features make poetry a powerful domain for empirical investigation of the mind–brain interface. Turner and Pöppel^4^ argued that poetry reflects and resonates with the hierarchical and rhythmic organisation of the human brain. Thus, neuroscientific explorations of poetry offer not only novel perspectives for neuroaesthetics but also valuable insights into broader questions concerning language, creativity, and emotion.

Compared to other art forms, poetry has received considerably less attention in cognitive neuroscience. Robust empirical work has focused on the neural mechanisms underlying music perception^5–7^, visual aesthetics^8–10^, and story comprehension^11–13^. In contrast, relatively few studies have examined how poetic language is processed in the brain^14,15^. Functional neuroimaging research indicates that reading poetry engages distinct neural circuits compared to prose, including lateral frontal, temporal, and occipital cortices, and regions associated with introspection and emotion^16,17^. Although these findings demonstrate that poetic language can activate emotional brain circuits, they offer only limited insight into the temporal dynamics of this engagement. In contrast to fMRI, M/EEG provides millisecond-level resolution, enabling a more detailed examination of the unfolding cognitive and emotional processes during the experience of poetry.

However, despite growing interest in the neurocognitive dimensions of literary language, there is currently a lack of open-access, high-temporal-resolution M/EEG datasets that allow for the systematic examination of poetic engagement. Existing public electrophysiological datasets in the domain of language largely focus on sentence comprehension^18,19^, emotion recognition^20,21^, or story/narrative comprehension^22,23^, but no publicly available dataset offers integrated behavioural and electrophysiological responses to authentic poetic stimuli.

To address this gap, we present a dataset comprising 64-channel EEG recordings and concurrent behavioural evaluations obtained during silent reading and contemplation on English-language Haiku and Senryu. These brief poetic forms are structurally constrained yet semantically and emotionally rich^24–26^. While Haiku tend to evoke natural imagery and seasonal transitions, Senryu focus on human experiences and emotional nuance, often delivered with irony or humour. Normative, traditionally structured Haiku and Senryu, with their highly constrained yet evocative structures, offer a unique balance of semantic richness and formal constraints, making them particularly well-suited for isolating aesthetic and cognitive responses to poetic language^27^; see also^28^. Their brevity not only facilitates precise experimental control but also foregrounds essential poetic features, such as imagery, affect and insight, that are central to neuroaesthetic inquiry.

Participants rated each poem along five dimensions: aesthetic appeal, vivid imagery, emotional impact, originality, and creativity. These were chosen to capture key facets of aesthetic and creative engagement with poetry. This dimensions were grounded in established theoretical frameworks in neuroaesthetics^3,14,29–31^ and empirical aesthetics^15,32–38^, which emphasize the role of evaluative, imagery-based, and affective processes in art perception and experience. The inclusion of originality and creativity reflects their centrality in psychological models^39–42^ of creative cognition and their empirical validation^43–48^. Further, these dimensions have recently been validated specifically in the context of poetry evaluation^49–52^. Together, the selected dimensions offer a theoretically grounded and empirically supported framework for capturing nuanced subjective responses to poetic stimuli.

This dataset captures not only neural responses to poetic language but also subjective evaluations of poetic quality, enabling the study of how aesthetic and creative judgments are instantiated in the brain. Further, the dataset also contains resting-state brain activity and several self-reported personality traits of the participants. The dataset complements and extends prior behavioural studies suggesting that creativity judgments in poetry are modulated by affective and perceptual dimensions^53,54^ and provides a foundation for linking such judgments to large-scale brain oscillations in future analyses. By making this dataset publicly available, we offer a novel, well-annotated resource that enables fine-grained exploration of how the human brain engages with poetic language. This resource will serve a wide community of researchers across cognitive neuroscience, psychology, linguistics, poetics, and digital humanities, and we hope it will stimulate further interdisciplinary research into the neurocognitive mechanisms of literary aesthetics and creativity.

## Methods

### Study design

This study employed a within-subjects design to investigate neural and behavioural responses to structurally concise but semantically rich poetic stimuli. Each participant completed several tasks: (i) completion of the demographic and personality questionnaires before the EEG recording, (ii) resting-state EEG recordings before and after the full session of poetic reading and evaluations, (iii) EEG recording during reading, contemplation and evaluation of poetic stimuli. Ethical approval was obtained from the Local Ethics Committee at the Department of Psychology, Goldsmiths University of London (Protocol Number: PS270423SCS). The experiment was conducted in accordance with the Declaration of Helsinki.

All participants provided informed content after reading an information sheet explaining the purpose of the study, its procedures, provisions for confidentiality, and intended use of the data. For example, the sheet explained: “This student research project aims to assess the neural signatures associated with perception and evaluation of creativity of poems. The study would explore how our brain responds while reading and contemplating poems. Here, you are to evaluate English poems based on your subjective feelings and experiences. No prior knowledge is expected.” Participants were explicitly informed about the use of their anonymized data for research and publication purposes (*e.g*., “All data collected from the study will be strictly confidential, stored on secure electronic information systems. All data that leaves the university will be anonymised, and names and other identifying information will be removed” and “The data will be used to further this research, any publication of the study will maintain confidentiality and anonymity of participants.”). Informed consent was obtained electronically prior to participation. Each participant completed an electronic consent form, where they confirmed (by selecting “Yes/No”) that they had read the Research Participant Information Sheet, understood the confidentiality provisions, and were informed of their right to terminate the experiment at any time without any consequence. They were also informed of their right to withdraw at any time, including post-study withdrawal via their unique participant ID. Only those participants who responded “Yes” to all mandatory items were allowed to proceed. Each participant then provided a unique ID *(“First three letters of your mother’s name followed by two digits of your month of birth”)* which was used in place of names. Participants were informed of their rights under the General Data Protection Regulation (GDPR) and Goldsmiths Research Guidelines, which outlined their rights regarding personal data (e.g., the right to be informed about data use and the right of access through subject access requests). All data were stored securely on university computers, and only fully anonymized data were exported for further analysis and sharing.

### Subject

Fifty-one right-handed individuals participated in the experiment (*N* = 51; 16 male, 28 female, 7 non-binary; mean age = 27.14 years, SD = 4.55). An *a priori* power analysis was conducted using G*Power 3.1^55^ to determine the minimum sample size required for a repeated-measures ANOVA with within-subject factors. Assuming an effect size of f = 0.229 (corresponding to η² = 0.05), α = 0.05, power = 0.80, three measurements (Haiku, Senryu, Control), a correlation of 0.5 among repeated measures, and a nonsphericity correction ε = 1, the required sample size was 33. The sample size of 51 participants exceeded this threshold, yielding an actual power of 0.81. All participants provided written informed consent and received monetary compensation (£30). However, four participants’ recordings (participant numbers # 5, 41, 42, and 46) were discarded during the spectral power validation analyses due to procedural interruptions (e.g., repeated absences), that compromised data integrity. Nevertheless, their EEG recordings are included in a separately shared dataset for transparency and potential reuse.

### Questionnaires

Before the main experiment, participants completed self-reported questionnaires on demographics, and personality traits. Mood was captured before and after the experiment. For each trait questionnaire, instructions were provided to participants, who chose the most appropriate option for each item. Participants were also informed that there were no right or wrong answers and were instructed to respond honestly and without long deliberation.

#### Demographic questionnaire

Demographic data comprised gender, age, ethnicity, location, education, and prior experience or exposure to poetry (e.g. “Do you like reading poetry?”, and “How long have you been associated with poetry?”). Of note, poetry liking was assessed with a single broad item (‘Do you like reading poetry?’), which was intended to capture general orientation toward poetry and did not distinguish between preferences for specific poetic traditions or genres.

#### Psychometric questionnaires

##### Positive and Negative Affect Schedule (PANAS)

This is a 20-item scale^56^ (10 positive, 10 negative) for assessing participants’ general affective tendencies over the past week. Example items include “Excited” and “Jittery.”

##### Openness/Intellect

It is a subscale of the Big Five Aspect Scale (BFAS^57^) to assess openness and intellect. It has 20 items, 10 items for assessing openness and 10 items for assessing intellect. Example items include “I love to reflect on things” and “I like to solve complex problems”.

##### Curiosity

It is a 10-item scale^58^ for assessing an individual’s tendency to seek out and explore novel information. Example items include “I enjoy learning about subjects which are unfamiliar”.

##### Vividness of Visual Imagery Questionnaire (VVIQ)

It is a 16-item scale^59^ to assess visual imagery, i.e., the ability to generate clear and detailed mental images. Participants were asked to imagine specific scenarios (e.g., a rising sun) and then rate the vividness of their mental images.

##### *Vividness of Auditory Imagery (BAIS-V*, termed here as *AVIQ*, auditory vividness imagery questionnaire, for clarity*)*

This 14-item scale^60^ evaluates the clarity and vividness of internally generated auditory imagery. Participants were asked to imagine specific auditory scenarios (e.g., attending a choir rehearsal) and then rate the vividness of their mental images.

##### Mindfulness Attention Awareness Scale (MAAS)

This 15-item scale^61^ provides an unidimensional measure of dispositional mindfulness and present-moment awareness. Sample items include “I rush through activities without being really attentive to them”.

##### Aesthetic Responsiveness Assessment (AReA)

This 12-item scale^62^ evaluates an individual’s sensitivity to aesthetic experiences in diverse artistic domains. Sample items include “I notice beauty when I look at art”.

### Stimuli

The stimulus set consisted of 210 short English-language texts: 70 Haiku, 70 Senryu, and 70 structurally matched non-poetic control texts. All Haiku and Senryu were sourced from prestigious literary competitions to ensure authenticity, quality, and adherence to traditional poetic forms. Specifically, poems were drawn from award-winning entries of the Haiku Society of America Haiku Award (1976–2022), the British Haiku Society Awards (2002–2021), and the Haiku Society of America Senryu Award (1988–2022).

Haiku and Senryu were chosen because they represent two minimalist Japanese poetic genres that share the same 3-line, 5–7–5 syllabic structure but differ thematically. Haiku traditionally evoke nature, seasonal references, and aesthetic imagery, while Senryu often focus on human affairs, irony, or humor^63,64^. This combination of structural uniformity (controlled syllabic constraints) and content variability (nature vs. human/social themes) makes them particularly suitable for controlled experimentation in cognitive and neuroaesthetic research. In the present study, we used original English-language Haiku (ELH) and Senryu rather than translated versions. ELH have been increasingly adopted in empirical investigations due to their brevity, structural consistency, and accessibility, enabling precise experimental control while engaging diverse cognitive processes such as imagery, emotion, and aesthetic evaluation^27,51–53,65,66^. The control texts were structurally matched to the Haiku and Senryu with a 3-line, 5-7-5 syllabic pattern, and were carefully selected and adapted from AI-generated repositories using prompts designed to avoid emotional depth, figurative language, or poetic elements. To confirm emotional neutrality, we conducted a sentiment analysis of the 70 control texts using the *sentimentr* package^67^ in R (v2.7.1), which showed that the corpus was, on average, neutral in polarity (mean = 0.17, SD = 0.37; range –0.82 to +1.25). These texts served as a structurally equivalent but semantically neutral baseline, allowing for isolating the effects of poetic content on neural and behavioural responses. See Table 1 for a typical example of each type of stimulus used in the study.

**Table 1:** Examples of the three stimulus categories used in the study: Haiku (nature-focused), Senryu (emotion-focused), and non-poetic control texts.

Haiku	Senryu	Control
harvest festival	refugee –	laptop powers up
jars of fig jam	where to bury	display glowing
full of galaxies	his child	ready for us

All stimuli adhered to the 3-line, 5-7-5 syllabic structure. Control texts were structurally matched but devoid of poetic devices, figurative language, or emotional content.

Importantly, all stimuli were presented without identifying information, including the poets’ names, award status, or genre classification (Haiku or Senryu). This anonymisation was implemented to minimise potential biases in creativity judgments, ensuring that evaluations were based solely on textual content. By eliminating contextual cues, our design sought to reduce familiarity effects, preconceived expectations, and prestige bias, thereby supporting a more objective appraisal of poetic aesthetics and creativity.

### Experimental procedure

The experiment was implemented using PsychoPy^68^, an open-source software platform widely used for the design of psychophysics and neuroscience experiments. The study employed a within-subjects, repeated-measures design in which each participant encountered all three stimulus conditions (Haiku, Senryu, and Control). The stimuli were presented in seven blocks of 30 trials each, totalling 210 trials (70 Haiku, 70 Senryu, 70 control). Each block comprised 10 stimuli of each type. The order of blocks and the trial sequence within each block were randomised per participant to mitigate order effects and ensure variability in presentation.

The EEG session began with a 5-minute resting-state period with eyes open, during which participants maintained visual fixation on a centrally presented cross. Following the initial resting-state period, the main experimental phase began, during which participants were presented with all three stimulus conditions: Haiku, Senryu, and control texts, along with on-screen instructions guiding them through the upcoming tasks.

**Fig. 1:**
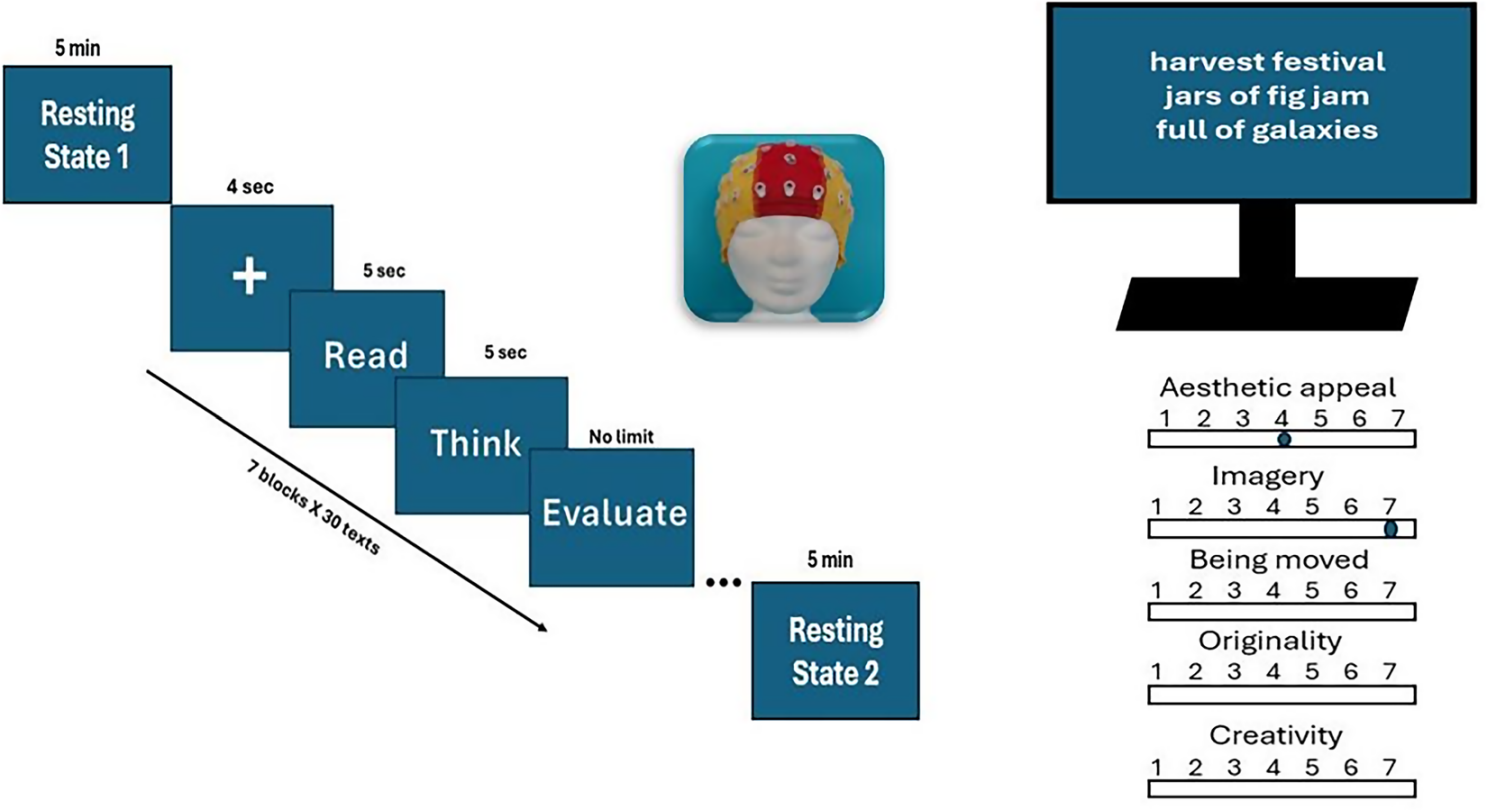
Experimental design. Each session began with a 5-minute resting-state EEG recording. Each trial consisted of a fixation cross (4 s), followed by poem presentation (5 s reading), during which the poem remained on screen for an additional 5 s contemplation period. In the subsequent evaluation phase, the poem was re-displayed on the left side of the screen together with the five rating scales (aesthetic appeal, vivid imagery, being moved, originality, creativity) presented on the right. The right column of the figure illustrates these elements together for compactness of visualization; in the actual experiment, however, they appeared sequentially in the order described. The session concluded with a second 5-minute resting-state recording.

In each trial, a stimulus was presented for 10 seconds: 5 seconds for silent reading and 5 seconds for contemplation, and these instructions (i.e. whether to read or to contemplate) were shown on the screen (see Fig. 1). Afterwards, participants rated the text on five dimensions using a 7-point Likert scale (1 = “very low,” 7 = “very high”): aesthetic appeal (“How aesthetically appealing is the poem?”), vivid imagery (“How vivid is the imagery?”), emotional impact (“How moved are you?”), originality (“How original is the poem?”), and creativity (“How creative is the poem?”). No time limit was imposed for making ratings. Participants initiated each new trial manually by pressing the space bar. Each poem remained on the screen during the reading and reflection phases, as well as during the subsequent evaluation phase, which had no time limit. This ensured that participants had sufficient time to contemplate and evaluate the text beyond the fixed reflection interval. The 5-second reflection represents a methodological compromise given the high number of trials (210), trial duration needed to be constrained to avoid excessive session length and associated participant fatigue or disengagement. Optional breaks were offered between blocks to reduce fatigue. Each poem was presented in its entirety (rather than word-by-word) to preserve a natural reading experience. Eye movements were monitored using electrooculographic (EOG) channels to enable artefact detection and correction.

A second 5-minute eyes-open resting-state period was recorded at the end of the session to complete the EEG protocol. Resting-states EEG was recorded in an eyes-open condition, during which participants were instructed to fixate on a central cross on the screen. Thus, choice was made to enhance ecological validity and ensure comparability with the visual and attentionally engaging nature of the main task (i.e. reading poetic texts). Further, eyes-open rest mitigates the dominance of occipital alpha typically observed in eyes-closed conditions^69^), thereby preserving spectral variability across bands. Prior studies also show that eyes-open rest engages neural networks more similar to task-based states^70,71^. Thus, this design provides a functionally aligned baseline for studying aesthetic and creative engagement.

### Data acquisition

Electroencephalographic (EEG) signals were recorded using a 64-channel active electrode system (BioSemi ActiveTwo; www.biosemi.com), with electrode placement conforming to the extended 10–20 international system. Eye movements were monitored using four electrooculographic (EOG) electrodes: two placed above and below the right eye (vertical EOG), and two at the outer canthi of both eyes (horizontal EOG). Two additional electrodes were placed on the left and right earlobes, whose average was used as the reference signal^72^. Electrocardiographic (ECG) activity was recorded using electrodes placed on the right collarbone and left waist. These signals were acquired as part of the recording setup but were not analyzed in the present study and are therefore not reported in the dataset description. All signals were recorded at a sampling rate of 512 Hz.

### Data preprocessing

EEG data were preprocessed using EEGLAB^73^. Signals were re-referenced to the average of the two earlobes. A 0.5 Hz high-pass filter was applied to remove slow drifts, and notch filters were applied at 50 Hz (±2 Hz) and 100 Hz (±2 Hz) to eliminate powerline interference. The continuous EEG data were segmented into epochs time-locked to stimulus onset. Each trial was epoched into a 15 s window spanning –4 to +11 s relative to stimulus onset. This included a 4 s pre-stimulus baseline, the 10 s stimulus period (5 s reading and 5 s contemplation), and a 1 s post-stimulus buffer to accommodate delayed neural responses (i.e. capturing those responses that might extend slightly beyond the visual presentation, like internal evaluation, imagery, memory access) and reduce edge effects in spectral analyses.

Independent component analysis (ICA) was performed using the EEGLAB^73^ function, runica(). ICA was applied to the 64 EEG channels (excluding external electrodes). Components associated with artefacts, such as eye blinks and muscle activity, were identified through a semi-automated procedure and removed following visual inspection. On average, one component was excluded per participant (M = 1.0, SD = 0.0), typically corresponding to blink artifacts. The resulting artefact-cleaned data were subsequently saved.

## Data Records

The datasets^74,75^ are available at OpenNeuro and contain data from 51 participants collected during the poetry assessment EEG study. The full collection comprises EEG and behavioural data from 51 participants, organized into two BIDS (Brain Imaging Data Structure)-compliant datasets. Poetry Assessment EEG Dataset 1^74^ includes 47 participants whose data were used in primary analyses, while Poetry Assessment EEG Dataset 2^75^ includes the four excluded participants (P105, P141, P142, and P146), whose EEG sessions were interrupted and later concatenated. These excluded participants’ data were not used in power spectral density analyses due to compromised recordings (e.g., repeated absence from sessions) but are shared for completeness and transparency. To maintain traceability while preserving anonymity, both datasets include a participants.tsv file that maps anonymized BIDS IDs (e.g., sub-001 to sub-047 in Poetry Assessment EEG Dataset 1^74^; sub-001 to sub-004 in Poetry Assessment EEG Dataset 2^75^) to the original participant identifiers used during data collection (e.g., P101–P151).

Each subject folder contains four core EEG files (channels.tsv, eeg.json, eeg.set, events.tsv), and a code/ directory includes the MATLAB preprocessing script (Preprocessing.m) and the BioSemi 64-channel coordinate file. Within the /derivatives/ directory, two subfolders—Behavioural_Ratings/ and Psychometric_Responses/—organize trial-level behavioural ratings (e.g., aesthetic appeal, imagery, emotional impact, originality, creativity) and trait-level psychometric measures (e.g., PANAS, openness, curiosity, VVIQ, AVIQ, MAAS, AReA), respectively.

The Behavioural_Ratings/ folder contains trial-level behavioural ratings for each participant, stored as individual.csv files (e.g., P101.csv). The Psychometric_Responses/ folder includes a single comprehensive.csv file containing demographic and trait-level psychometric measures for all participants along with references to the source questionnaires. Stimulus materials (all 210 poems and block-wise assignments) are provided in a /stimuli/ folder. Each dataset is accompanied by a comprehensive README file documenting structure, variable definitions, preprocessing steps, and usage instructions, with additional README files in the behavioural and psychometric subfolders. To facilitate navigation, Table 2 provides a structured overview of the dataset contents and file organization, including folder hierarchy, file types, and descriptions. This table summarizes the location of raw EEG data, event metadata, behavioural and psychometric responses, questionnaires, stimuli, preprocessing code, participant mappings, and documentation files.

**Table 2:** Overview of dataset contents and file structure, including folder hierarchy, file types, and descriptions of all components: raw EEG data, event metadata, behavioural and psychometric responses, questionnaires, stimuli, preprocessing code, participant mappings, and documentation files.

File Type(s)	Description
**Poetry Assessment EEG Dataset 1**^74^ **(47 participants’ dataset)**	Raw EEG data for 47 participants, organized in BIDS format.
**Poetry Assessment EEG Dataset 2**^75^ **(4 excluded participants’ dataset)**	Raw EEG data for 4 participants, organized in BIDS format.
**Each Folder contains the following folders/files**	
code/	
Preprocessing.m	MATLAB preprocessing script.
BioSemi64.loc	BioSemi 64-channel electrode coordinate file.
derivatives/Behavioural_Ratings/	
PXXX.csv	Trial-level behavioural ratings (PoemName, PoemType, Block, 5 rating scales).
README_Behavioural	Documentation of behavioural data structure and usage notes.
derivatives/Psychometric_Responses/	
Psychometric_Responses.csv	Demographic and psychometric data.
.pdf	Questionnaire references and demographic questions.
README_Psychometric	Description of questionnaire variables and scoring.
stimuli/	Stimuli information
.pdf	All 210 texts (Haiku, Senryu, Control)
.xlsx	Trial assignment files for Block_1 to Block_7.
sub-001 to sub-047	Each subject folder contains:
sub-***_task-readpoetry_channels.tsv	Channel information for EEG data.
sub-***_task-readpoetry_eeg.json	EEG metadata.
sub-***_task-readpoetry_eeg.set	EEG data in EEGLAB format.
sub-***_task-readpoetry_events.tsv	Trial-by-trial event annotations.
Top-level files	
dataset_description.json	Dataset metadata file.
participants.tsv	Participant information and demographics.
README	Comprehensive documentation of dataset structure.
task-readpoetry_events.json	Key for experimental event codes and descriptions.

All EEG, behavioural, and psychometric data were anonymized. Participant identifiers were coded (P101–P151), and no names, dates of birth, or other direct identifiers are included.

Of note, the anonymized participant IDs (e.g., PXXX) are used consistently across all data modalities, enabling reliable cross-referencing between EEG data, behavioural ratings, and psychometric responses. Resting-state recordings collected at the beginning and end of the session are included in the raw EEG files. These segments can be identified via event codes 65285 and 65286 (start) and 65287 and 65288 (end) in the corresponding events.tsv files.

## Technical Validation

We implemented strong quality control protocols across all modalities to ensure the reliability and scientific utility of the dataset.

### Behavioural Validation

To verify task compliance and engagement, we analyzed behavioural ratings for scale use and consistency across conditions. Participants used the full range of all five 7-point dimensions (aesthetic appeal, imagery, emotional impact, originality, creativity), indicating appropriate engagement. Internal consistency of trait questionnaires was high (Cronbach’s α > 0.80 across all scales), and response completeness was 100%. To examine potential fatigue effects, we computed block-level means across the seven experimental blocks for each evaluative dimension. Ratings remained stable over time (e.g., Aesthetic Appeal: M = 4.04, SD = 0.10; Imagery: M = 4.65, SD = 0.15; Emotional impact (‘Moved’): M = 3.70, SD = 0.10; Originality: M = 3.95, SD = 0.07; and Creativity: M = 3.92, SD = 0.08), and linear trend analyses revealed all slope estimates were near zero and no significant changes across blocks (all *p*s > .50), indicating no systematic decline in ratings over time.

**Fig. 2:**
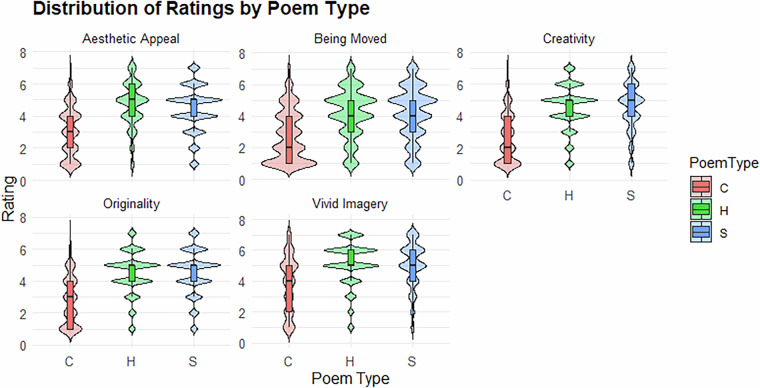
Distribution of ratings across three conditions (C = Control, H = Haiku, and S = Senryu) for five evaluative dimensions: aesthetic appeal, imagery, emotional impact (being moved), originality, and creativity. There are five subplots, one for each evaluative dimension. Each subplot shows three violin plots depicting the full distribution of ratings across the three conditions for that specific evaluative dimension.

The mean ratings (see Fig. 2) showed that Haiku received the highest aesthetic appeal score (M = 4.68), followed by Senryu (M = 4.40), while control texts were rated lower (M = 3.04). Haiku was also rated highest for vivid imagery (M = 5.12), slightly above Senryu (M = 5.00), with controls scoring lower (M = 3.81). For emotional impact (‘Moved’), Senryu (M = 4.18) slightly outperformed Haiku (M = 4.16), while control texts again scored the lowest (M = 2.73). For originality, ratings were close between Haiku (M = 4.50) and Senryu (M = 4.52), both surpassing controls (M = 2.79). For creativity, ratings were similarly high for Haiku (M = 4.54) and Senryu (M = 4.53), with controls rated notably lower (M = 2.65). Together, these descriptive differences indicate that the poetic stimuli elicited stronger aesthetic and creative evaluations than control texts, validating the behavioural task. These results indicated that while Haiku was more aesthetically appealing, Senryu was perceived as slightly more emotionally moving, with both poetic forms being rated highly for vividness and originality. A detailed analysis of these behavioral findings has been reported in a recent study^51^.

### EEG Validation

Spectral power analyses were conducted across five canonical frequency bands (delta: 0–4 Hz, theta: 4–8 Hz, alpha: 8–12 Hz, beta: 12–30 Hz, gamma: 30–48 Hz) using Welch’s method^76,77^. Four participants’ recordings were excluded due to procedural interruptions that compromised data integrity (e.g., repeated absence from recording sessions), resulting in a final sample of 47 participants (13 male, 28 female, 6 non-binary; mean age = 27.06 years, SD = 4.66).

Power spectral density (PSD) was calculated with the 10-s post-stimulus period divided into 2-s windows (500 ms overlap). Periodograms were computed per electrode and trial, with the first 4 seconds (–4–0 s) serving as baseline, 0–5 s as the early (reading) phase, and 5–10 s as the late (contemplation) phase. Spectral power values were averaged across frequencies within a frequency band, log-transformed, and normalized by baseline power to account for inter-individual variability.

**Fig. 3:**
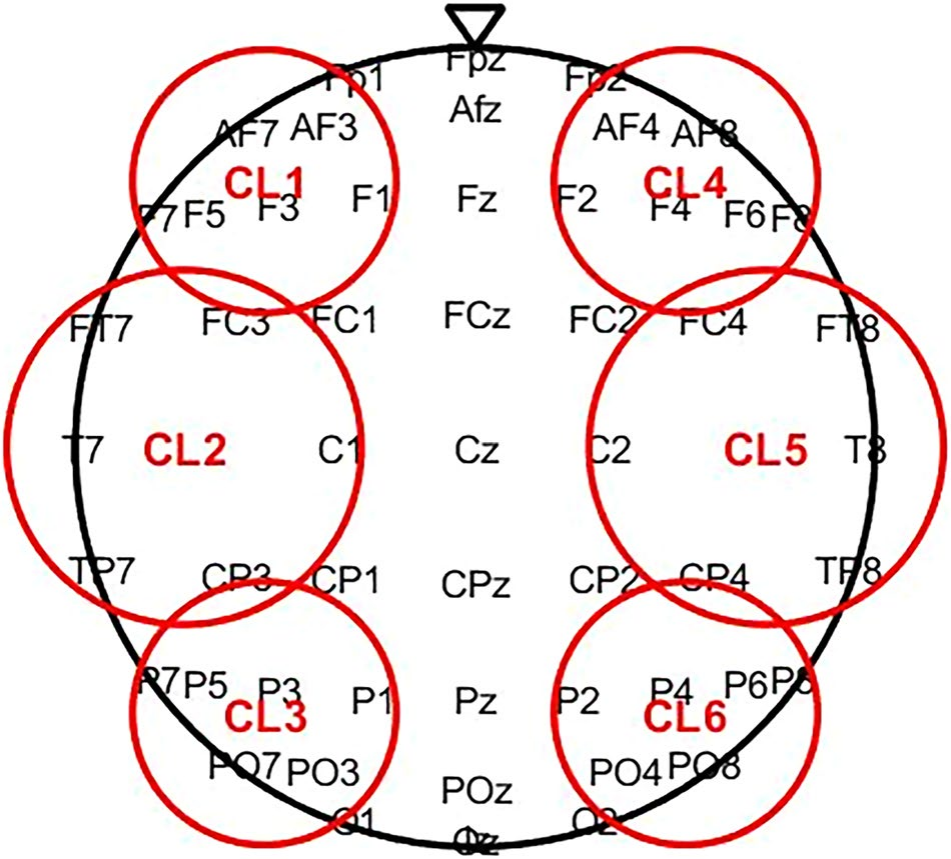
Electrode groupings into six scalp-based regions of interest (ROIs) used for spectral quality checks. Electrodes were clustered according to the international 10–20 system into frontal, fronto-temporal, and parieto-occipital regions, separately for the left (CL1–CL3) and right (CL4–CL6) hemispheres. This organization provides region-specific coverage for assessing EEG signal quality across the scalp.

To ensure cortical region-specific coverage of neural activity, and in alignment with standard practices in EEG research^78–81^, we grouped electrodes into six scalp-based clusters (frontal, fronto-temporal, and parieto-occipital in each hemisphere; see Fig. 3) to provide broad anterior–posterior coverage while keeping the structure simple and interpretable. This clustering scheme was based on topography alone, rather than frequency- or power-specific characteristics. Mid-line electrodes (e.g., FCz, Cz, Pz) were not grouped into a separate ROI, as they often capture spatially averaged or overlapping activity from adjacent electrode regions, making them less suitable for coarse regional grouping in exploratory analyses. This scheme was intended to balance spatial coverage with interpretability, and our primary goal was to demonstrate that the dataset supports interpretable frequency-domain analyses rather than to test specific hypotheses about regional specialization.

**Fig. 4:**
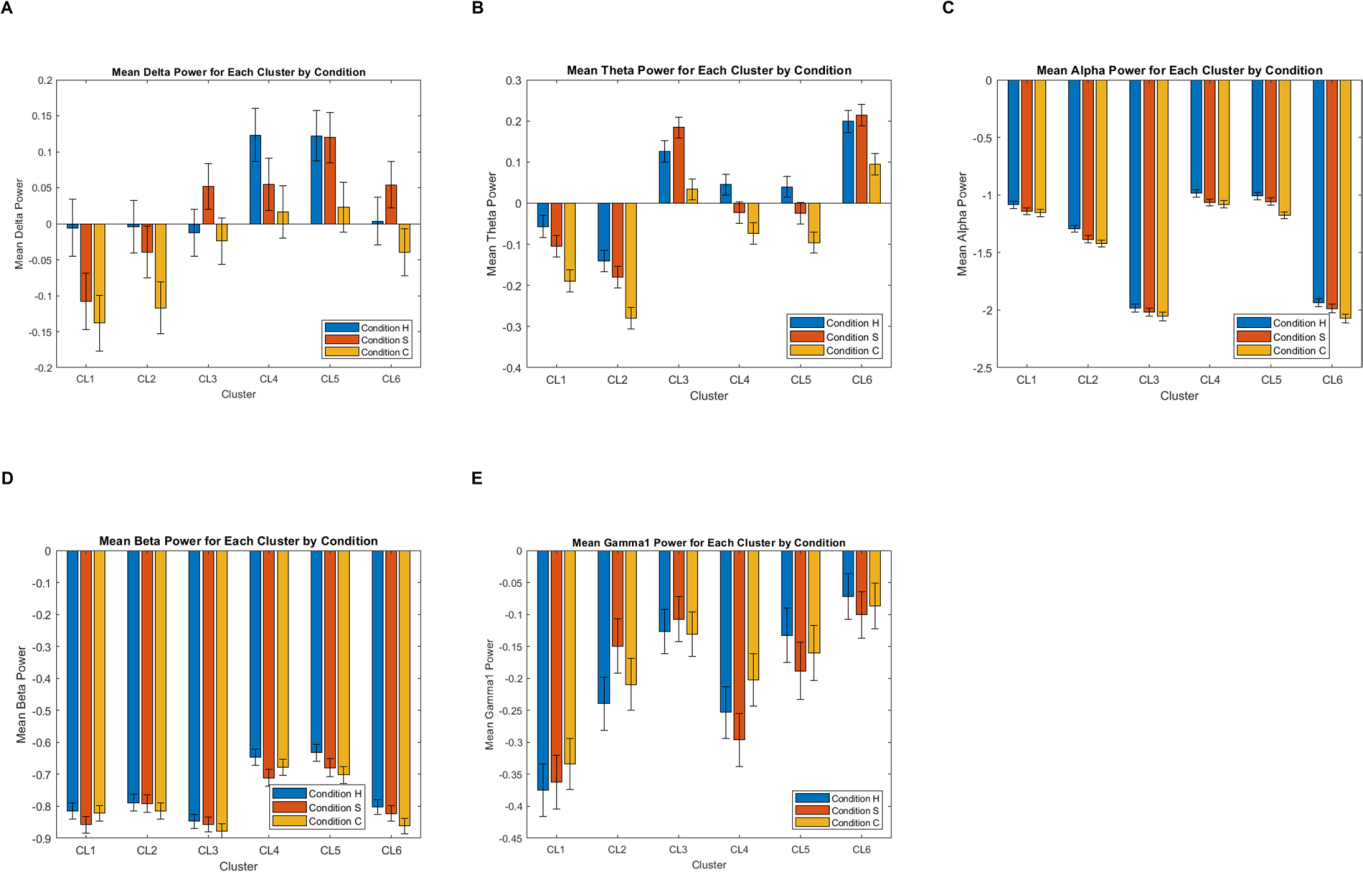
Power spectral density (PSD) estimates across all five canonical frequency bands (delta, theta, alpha, beta, gamma) and electrode clusters. The plots illustrate that robust and reliable neural oscillations were obtained across participants, confirming that the dataset supports frequency-domain EEG analyses. Error bars represent standard error of the mean.

**Fig. 5:**
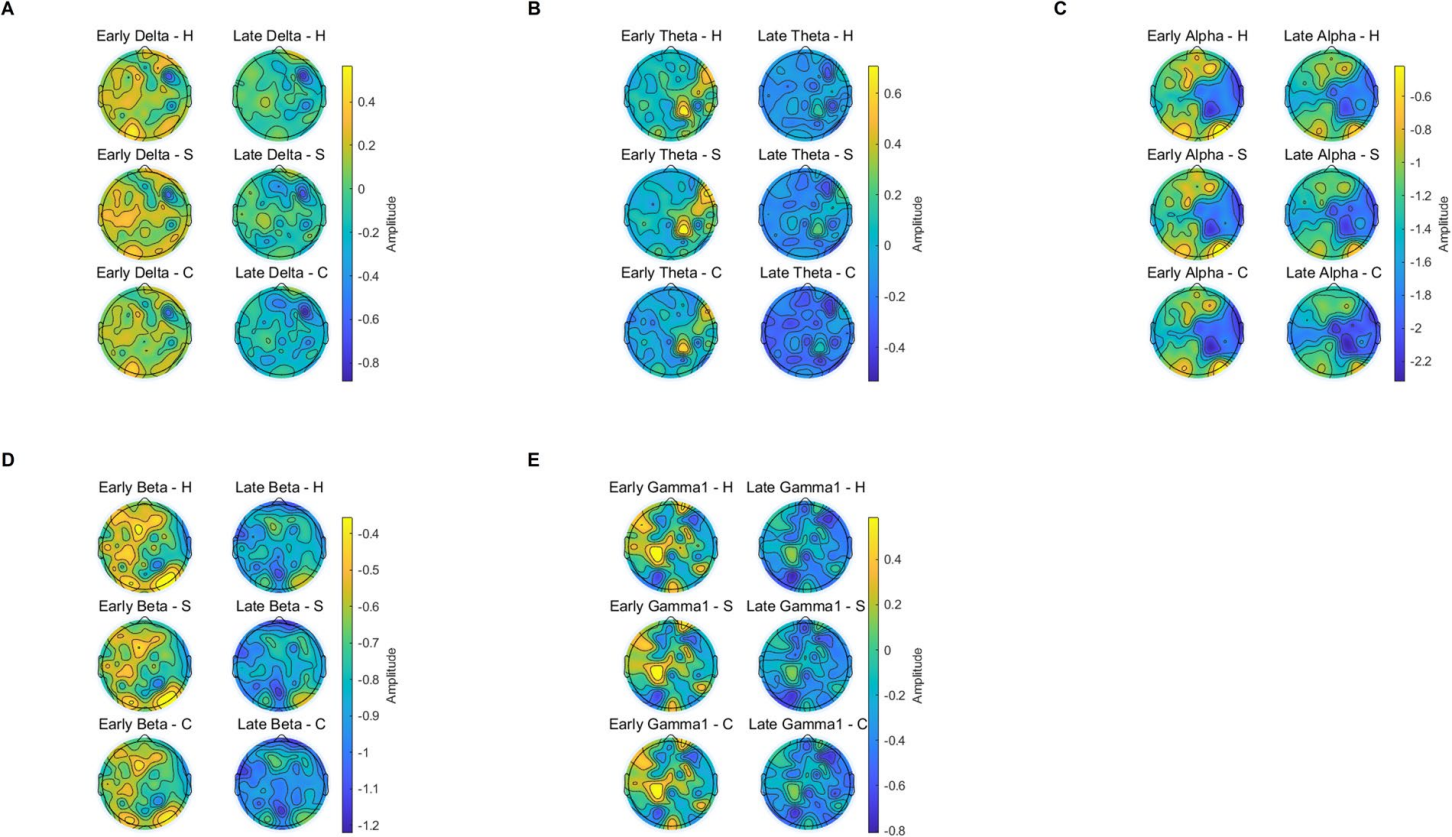
Topographical maps of EEG power density across the five canonical frequency bands (delta, theta, alpha, beta, gamma) for Haiku, Senryu, and Control conditions during early (0–5 s) and late (5–10 s) task intervals. These maps are presented as descriptive illustrations of scalp-level power distributions and serve as validation of spatial resolution and signal quality. No inferential comparisons between conditions are reported, in line with the journal’s guidelines. While visual inspection suggests similarities (e.g., between Haiku and Senryu), formal statistical evaluation requires additional analysis (e.g., cluster-based permutation tests), which the dataset fully enables but are beyond the scope of this descriptive report.

To provide a descriptive overview of EEG spectral responses, Fig. 4 shows mean power values across six electrode clusters for each frequency band (delta, theta, alpha, beta, gamma) and condition (Haiku, Senryu, Control). These plots illustrate that reliable oscillatory activity was obtained across clusters and conditions, with visible variation across bands and hemispheric groupings. Error bars represent the standard error of the mean, confirming stable estimation across participants. To provide a descriptive overview of the spatial distribution of spectral power, Fig. 5 presents topographical maps for all five canonical frequency bands (delta, theta, alpha, beta, gamma) across Haiku, Senryu, and Control conditions at early and late intervals. These scalp distributions are shown without inferential contrasts (in line with the journal’s guidelines), and serve to illustrate the spatial resolution and signal quality of the dataset. The maps confirm that robust and interpretable topographical patterns are observable across conditions, supporting the dataset’s suitability for frequency-domain and topographical analyses.

Together, PSD plots (Fig. 4) and topographical maps (Fig. 5) confirm that the dataset preserves meaningful oscillatory and spatial organization. In combination with behavioural compliance checks (e.g., full scale use, systematic stimulus differentiation), these validations indicate that the EEG signals are of high quality and suitable for a wide range of advanced applications such as connectivity analysis, network modeling, and machine-learning approaches. These quality assurances enable a wide range of downstream applications, supporting both conventional and cutting-edge EEG analysis.

## Usage Notes

The datasets^74,75^ are provided in standard EEG formats compatible with widely used analysis toolboxes, including EEGLAB, FieldTrip, and MNE-Python. Researchers may choose to apply their own preprocessing pipelines depending on the analytical goals. While basic preprocessing guidance is described in the Methods section, users may wish to adapt alternative strategies to suit their specific hypotheses. This study supports multiple lines of research. First, it enables trial-level investigations of brain-behaviour relationships, linking EEG dynamics (spectral, connectivity, network parameters etc) with subjective evaluations of poetic stimuli. Second, the inclusion of demographic and psychometric data allows for analysis of individual differences in aesthetic and creative evaluation. Third, the data can also be used for methodological development, including EEG processing, and machine learning-based decoding of aesthetic or creative judgments. Finally, the availability of both resting-state and task-based EEG supports comparative studies of intrinsic and stimulus-driven neural dynamics. However, given the modest sample size, users are advised to interpret group-level inferences with appropriate caution. Nevertheless, the dataset provides a valuable resource for advancing research in neuroaesthetics, cognitive poetics, and the neuroscience of creativity.

## Data Availability

The datasets^74,75^ are available at OpenNeuro and contain data from 51 participants collected during the poetry assessment EEG study. The full collection comprises EEG and behavioural data from 51 participants, organized into two BIDS (Brain Imaging Data Structure)-compliant datasets. Poetry Assessment EEG Dataset 1^74^ includes 47 participants whose data were used in primary analyses, while Poetry Assessment EEG Dataset 2^75^ includes the four excluded participants, whose EEG sessions were interrupted and later concatenated. These excluded participants’ data were not used in power spectral density analyses due to compromised recordings (e.g., repeated absence from sessions) but are shared for completeness and transparency.

## Code availability

All MATLAB preprocessing code is included in the code/ directory of both Poetry Assessment EEG Dataset 1^74^ and Poetry Assessment EEG Dataset 2^75^, available at OpenNeuro. Each dataset contains an identical version of the script Preprocessing.m, which documents the complete EEG preprocessing pipeline applied to the raw data.
